# The Vaccine Communication Demands on Community-Based Workforces

**DOI:** 10.3389/fpubh.2022.827378

**Published:** 2022-02-04

**Authors:** Lauren Rauh, Dave Patry, Michelle Zambrano, Hannah Stuart Lathan, Emilio Tavarez, Ayman El-Mohandes

**Affiliations:** ^1^City University of New York (CUNY) Graduate School of Public Health & Health Policy, New York, NY, United States; ^2^Hunger Free NYC, New York, NY, United States; ^3^Health Leads, New York, NY, United States

**Keywords:** vaccine promotion, health communication, trust in source, COVID-19, community workforces

## Abstract

Community-based organizations (CBOs) are experiencing some of the highest demand in years for a wide spectrum of health and social services. Their client-facing employees have taken on a new, challenging role as a sought-after source of COVID-19 vaccine information and guidance. These workforces operating on the frontlines, do their best to meet the increased need for services and information, often without additional resources or training to do so. The most effective immediate response to this challenge is a comprehensive communication support system working in tandem with CBOs. Our three organizations, the New York Vaccine Literacy Campaign at the CUNY Graduate School of Public Health and Health Policy, Hunger Free NYC, and Health Leads, have collaborated in key short-term approaches to meet these needs. We outline these processes and anticipated outcomes and offer lessons learned to advocate for long-term structural changes needed to increase community-level communication support.

## Introduction

Community-based workforces are the most connected to the priorities of populations they serve, building trusted relationships over time through one-on-one interactions and by being a reliably accessible and available presence. Leveraging this trust, these workforces are a much-needed resource to the underserved and now fill, to the best of their abilities, a gap in appropriately tailored COVID-19 communication and information resources. While these organizations have adjusted and adapted to new modes of delivery throughout the pandemic, many find themselves responding to requests to reinforce public health advice that is outside of their training and scope of work, namely, COVID-19 vaccine information.

Through partnerships with community-based organizations (CBOs), the New York Vaccine Literacy Campaign (NY VLC) (1) at the CUNY Graduate School of Public Health and Health Policy (CUNY SPH) has aimed to mitigate the demands of COVID-19 vaccine communication heaped upon trusted frontline workforces. Two such partnerships with the NYC branches of Health Leads (2) (HLNY) and Hunger Free (HFNYC) (3) are described here to demonstrate what has been lacking, how communication needs can be fulfilled effectively, and implications for minimizing the overburdening of an already overextended workforce.

The health and social service systems in NYC, and across the United States broadly, lacked a unified and efficient communication infrastructure well before the pandemic, leading to widespread public confusion at the onset of this crisis. Even now, individuals with remaining questions and concerns about the COVID-19 vaccines must make decisions about which messages and messengers to trust in the face of conflicting and confusing information. Meanwhile, the entrenched trust that organizations like Hunger Free America and Health Leads have developed through their years of service continue to be underestimated and underfunded throughout the response to the pandemic. We have developed strategies to champion the responsibility of the “trusted vaccine messenger” and have begun thinking forward as to how to build community-level trust in vaccination more broadly by addressing identified communication gaps and challenges, especially among the most under-resourced communities.

## Who We Are

*HFNYC* is the New York City affiliate of Hunger Free America, a national, nonpartisan organization working to enact the policies and programs needed to end domestic hunger and ensure that all Americans have sufficient access to nutritious food. In NYC, the organization operates the Food Action Board Program, which develops community members' leadership, advocacy, and community organizing skills to promote food security and economic opportunity within their communities. Additionally, the Benefits Access (BA) team assists individuals through every step of the SNAP and WIC application processes. A trained BA specialist works with individuals to determine eligibility, ensure qualification for benefits, and process all requisite documentation. During the early stages of the COVID-19 pandemic, the NYC Department of Health and Mental Hygiene broadened the scope of the grant under which the BA team operated, allowing the team to expand to all five boroughs to meet the demand of increased incoming referrals.

*Health Leads* is a national innovation hub that unearths and addresses the deep societal roots of racial inequity that affect health and creates smarter, more equitable, community-centric, and connected public health and essential resource systems. During the pandemic, Health Leads launched a two-year (2020–2022). Respond and Rebuild Strategy to help communities meet urgent resource demands worsened by the COVID-19 pandemic, while also redesigning and rebuilding the systems necessary for a more equitable and crisis-resilient future. Health Leads is a founding partner of the Vaccine Equity Cooperative, (4) which works to ensure racial equity in COVID-19 vaccine development and distribution by reinforcing and supporting CBOs, including public health departments, with advocacy, funding, and informational resources. This initiative supports the rebuilding of public trust that is necessary to address long-term disparities and prepare for future crises.

Building upon its commitment to engage New Yorkers to understand how COVID-19 has impacted their lives, a team of faculty and staff at CUNY SPH launched the NY VLC in April 2021. The initiative supports community-based, affiliated, and direct service organizations in their roles by increasing community-level access to vaccine education and information through tailored webinars, education modules, training, and other capacity-building resources.

## In Nyc, a Spike in Demand for Services, a Pivot For CBO Workforces

While some services saw a reduction in patronage during the pandemic, either due to limited availability or a reprioritization due to safety or other immediate life concerns [e.g., fewer individuals sought health care in 2020 (5)], many demands for supportive resources skyrocketed. More people turned to organizations in their communities as a safety net to help them meet their basic life needs. And while information about the pandemic and how to stay safe has since become more stable, those early desperate days were chaotic for many. In our qualitative research (6, 7) individuals described an information environment that left them feeling less certain of what and whom to believe. Communities looked to the providers/organizations with whom they developed historically consistent, trustworthy, and solution-oriented relationships for reliable information on topical areas that these organizations did not regularly provide.

The HFNYC's Benefits Access (BA) team saw a 67% increase in SNAP and WIC household pre-screenings from 2019 to 2020. As a result, they connected 56% more food insecure New Yorkers to benefits. Their work generated approximately a total of $11,391,000 in annual SNAP benefits and $33,955.68 in WIC benefits. While in-person work was put on pause, their work continued virtually and—unbeknownst to the team at the time—desperate parents circulated their contact information throughout the public school system.

To reduce caseloads for BA team members, the organization sought partnerships with other CBOs to quickly disseminate critical information about federal nutrition programs, P-EBT and the Get Food NYC program. HFNYC came to be known as a “high trust” organization in many communities, and BA team members were engaged for assistance beyond nutrition programs, fielding questions related to other social services, add even COVID-19 vaccines. In September 2021, the Human Resources and Services Administration (HRSA), a federal contracting agency, awarded HFNYC with a grant to conduct COVID-19 outreach in coordination with its existing nutrition outreach efforts. HFNYC hired a team of community organizers, many of them clients, to work across the South Bronx, Central Queens, and Central Brooklyn to provide information to communities with low vaccination rates.

HLNY's Maternal Health Initiative aims to improve maternal health outcomes for Black women in NYC by improving access to benefits, removing barriers to accessing resources, increasing utilization of benefits, and empowering caregivers to support individuals through their pregnancy and postpartum experience. HLNY works collaboratively with communities most affected by maternal health inequities to improve health outcomes for pregnant and birthing people and to support CBOs in their capacity to connect all pregnant/postpartum clients with necessary resources to be healthy. In response to COVID-19, HLNY adjusted the efforts on the Maternal Health Initiative to align with the organization's overarching Respond & Rebuild strategy. The adjusted priorities included convening partners to support coordination of resources and strengthen referral pathways, supporting maternal health caregiver sustainability, and connecting pregnant and new parents to emergency food.

During this time, HLNY continued to build and strengthen the network of maternal health partners in Central Brooklyn and the Bronx. While these maternal health providers are accustomed to discussing medical questions and concerns, many found themselves unprepared to address the high levels of concern and gaps in knowledge expressed by their patients about the COVID-19 vaccines, often fueled by densely propagated misinformation. When asked about perceived barriers to vaccination, most maternal health providers reported that clients were reluctant to discuss COVID-19 vaccination and often cited barriers such as lack of childcare as the reason they remained unvaccinated, even if it was not the main reason for their hesitation.

### Supporting CBOs Through Capacity-Building and Resource Co-design

HFNYC and HLNY recognized the urgency for targeted support for their employees and partners newly entrusted to provide vaccine information by the communities they served and worked closely with the NY VLC to develop two distinct approaches to address this challenge. We describe the two approaches below.

#### Hunger Free NYC

First, HFNYC and the NY VLC developed a suite of educational materials related to COVID-19 and the vaccines, targeting specific information requests (i.e., adults over the age of 85 years old). In addition, due to the high volume of requests being fielded by pregnant and postpartum clients, HFNYC sought to develop a more comprehensive effort to address the concerns of this population. Their primary, near-term goal was to equip their BA staff with a toolkit to use in one-on-one interactions with clients. The NY VLC and HFNYC teams developed the toolkit, complete with information on COVID-19 infection risk, particularly to pregnant people, benefits of vaccination, understanding potential vaccine side effects, a stepwise guide to vaccine appointments, and a script geared toward anticipating various questions. This collaboration also led to the production of an informational virtual panel discussion to answer questions from pregnant and breastfeeding attendees.

These resources put the necessary tools into the hands of client-facing CBO employees, best positioned to communicate critical public health information. To use these materials effectively, employees will receive comprehensive training on both the material itself as well as strategies to communicate such health messages to clients. Through this process, CBO employees will become equipped to deliver effective support services around COVID-19, reducing the amount of time and effort spent combating misinformation, maintain trust with their clients, and refocus their efforts to their primary goal of reducing food insecurity and other structural inequities. HFNYC will also work to disseminate these materials so that community leaders and partner organizations may use these tools to facilitate conversations with their own clients.

#### Health Leads New York

The HLNY team and NY VLC began discussing a partnership in spring 2021 as the COVID-19 vaccine became more available. HLNY has long-established, close relationships with maternal health CBOs and saw the opportunity to co-design strategies to provide these partners with better resources to address their clients' concerns about the COVID-19 vaccine. The NY VLC and HLNY teams collaborated on a survey for CBO partners to identify their biggest communication challenges. The survey results suggested that respondents overwhelmingly lacked adequate resources to address their maternal health clients' questions and apprehensions. Maternal health providers reported numerous perceived barriers and concerns, which were coupled with existing and often exacerbated unmet social needs due to the pandemic. HLNY organized a virtual group co-design session to further determine the issues affecting vaccine confidence at the community level and how these challenges could be addressed in a way sensitive to community priorities. Through iterations of brainstorming and discussion, the focus group homed in on key issues, including resolving childcare needs for parents to allow them to attend a vaccine appointment; and addressing the need for trusted community members to disseminate accurate vaccine information.

In the second virtual group session, HLNY worked with participants to ideate solutions to the problems defined in the first session. A community member who attended both sessions shared why this approach to generating solutions mattered to her, saying, “It is really important that we included the clients because sometimes you think you understand something when making decisions or strategies. It is really important to include people who are “going through it” in the process.” (Participant 1, Co-design session 2, 11/5/2021). One provider stated that by participating in the design sessions and collaborating with others, she felt that, “I have more tools in my toolbelt to help others.” (Participant 2, Co-design session 2, 11/5/2021).

Next steps include producing one to two innovative solutions and a set of recommendations for participating CBOs. HLNY is currently gathering input on the feasibility and potential impact of two ideas generated during the sessions, which focus on peer community members as trusted messengers for pregnant and postpartum people: (1) Recruit community members who are pregnant or new parents to create video testimonials that explain motivations to get the COVID-19 vaccine and their vaccination experience; share these videos with maternal health clients in community or clinical settings. Video testimonials have been used widely in public health campaigns (8). (2) Expand upon the peer-to-peer support model for pregnant and postpartum community members to be “vaccine ambassadors”. Vaccine ambassadors would be trained, compensated, and provided resources to have one-on-one conversations and provide follow up support with maternal health clients who express COVID-19 vaccine hesitancy. Peer-to-peer models have been successful in other areas of maternal health for this priority population, including breastfeeding peer counselors for WIC participants (9) and group prenatal care models such as Centering Pregnancy (10). Vaccine ambassador programs in many states have successfully increased COVID-19 vaccination rates (11). There is a need to prioritize maternal health patients and new parents to address the unique concerns of their peers and share their vaccine experiences and outcomes as ambassadors.

## Discussion: Sustaining the CAPACITY of CBO Workforces as Trusted Messengers

When tasked with a risk-benefit analysis, i.e., the decision to accept COVID-19 vaccination, underserved community members turned to the organizations that have consistently provided support. It is important to recognize this first as an indication that community-based workforces like HFNYC and Health Leads' partners are regarded as most reliable at meeting the needs of their communities, also important, that the larger systems continue to fail the communities that consistently experience, and frequently expect, a vacuum of resources. We see the communication system breakdown coupled with low levels of vaccine literacy (12)—the societal and environmental factors that increase individual and community knowledge, access, and confidence in vaccination—in vulnerable communities as a direct outgrowth of structural inequities that predate the pandemic. Vigorous, well-funded anti-vaccination campaigns compound this by pushing political and ideological beliefs at odds with pragmatic, science-based preventive measures needed to stop the spread of the virus. In winter 2022, two years into the pandemic, these issues persist and may bear some responsibility for the 17% individuals in NYC who steadily remain unvaccinated (13). The communities with unanswered vaccine questions and misinformation-fueled concerns are largely the same communities that are generally underserved. These are communities with pressing life priorities like fear of eviction, food insecurity, and barriers to affordable health care. National (14) and local (15) survey data from September 2021 showed the most powerful predictor of remaining unvaccinated was lack of health insurance, despite the vaccine offered free of charge; this may be a surrogate for other associated unfulfilled needs.

Creating and maintaining a new cadre of “vaccine communicators” within most CBOs is unsustainable—financially, organizationally, and socially—in service environments with traditionally overextended resources. Given the rapid mutation of the virus over the last 2 years, COVID-19 vaccination may remain a top concern and will continue to overlap with other urgent health or social issues. However, the funding released to meet this crisis is running out. A resilient communication infrastructure could be developed and sustained through policy solutions such as the HOPE Act (16), which would leverage client-facing technology to coordinate access to multiple government agencies working on anti-poverty, health care, nutrition, housing, work support programs, and nonprofit aid for low-income Americans. This would allow people access all the support systems they are eligible for at once, instead of having to apply individually to each agency. Additionally, assessing the continuation of funding streams created by the CARES Act and other similar emergency actions could maintain the capacity afforded these workforces during the pandemic (17). Newly formed communication channels would be always active, not just in health emergencies, and reinforced by robust social services to facilitate and maintain a bottom-up feedback loop.

In the face of multiple, amplified, and intersecting health and social needs, community-based organizations cannot assume the principal role of health communicators without substantial sustained investment. Restructuring policies and funding mechanisms to center communities and the workforces that serve them, would strengthen the capacity needed to meet demand and solidify a distinct community-driven communication system for the long-term. Throughout, and before the pandemic, client-facing, community-based workforces have effectively provided tailored messaging and training to those who need it most. The federal, state, and local agencies responsible for developing and disseminating key public health information must fund these workforces as key players in the long-term pandemic response to ensure their continued capacity to act as trusted messengers.

## Author Contributions

LR and HL: manuscript conception and design. LR, DP, MZ, HL, ET, and AE-M: draft manuscript preparation. All authors reviewed and approved the final version of the manuscript.

## Funding

The New York Vaccine Literacy Campaign is supported by the New York Community Trust, the Altman Foundation, the New York State Health Foundation, and the Samuel Freeman Charitable Trust.

## Conflict of Interest

The authors declare that the research was conducted in the absence of any commercial or financial relationships that could be construed as a potential conflict of interest.

## Publisher's Note

All claims expressed in this article are solely those of the authors and do not necessarily represent those of their affiliated organizations, or those of the publisher, the editors and the reviewers. Any product that may be evaluated in this article, or claim that may be made by its manufacturer, is not guaranteed or endorsed by the publisher.
